# Supplementary material for: How frequent is routine use of probiotics in UK neonatal units?
Source: BMJ Paediatr Open. 2023 Jul 14;7(1):e002012. doi: 10.1136/bmjpo-2023-002012 (PMC10351264; doi:10.1136/bmjpo-2023-002012)
Supplement: Supplementary data [file bmjpo-2023-002012supp001.pdf]

Evaluating the use of probiotics in UK neonatal units: a national, trainee-led survey

This is a national, trainee-led survey evaluating the use of probiotics in all UK neonatal units, conducted as part of NeoTRIPS.

NeoTRIPS is a national neonatal study group, using the power of trainees to improve neonatal care through large scale quality improvement and research projects.

Please ensure that only 1 trainee responds per neonatal unit.

* Required

1. What is the name of your neonatal operational delivery network (ODN)? *

Mark only one oval.

- East Midlands Neonatal ODN
- East of England Neonatal ODN
- Neonatal Network Northern Ireland
- North Central and East London Neonatal Network
- North West London Neonatal ODN
- North West Neonatal ODN
- Northern Neonatal Network
- Scottish Perinatal Network
- South East Coast Neonatal Network
- South London Network
- South West Neonatal Network
- Thames Valley & Wessex Neonatal Network
- Wales Maternity and Neonatal Network
- West Midlands Neonatal ODN
- Yorkshire & Humber Neonatal ODN

2. What is the name of your neonatal unit/local hospital? *

____

3. What is the level of your neonatal unit? *

Mark only one oval.

- Level 1
- Level 2
- Level 3

4. Does your neonatal unit currently use probiotics? *

Mark only one oval.

- ( ) Yes *Skip to question 5*
- ( ) No *Skip to question 20*
- ( ) Don't know

Neonatal units utilising probiotics

5. What probiotic do you currently use? *

Mark only one oval.

- ( ) ProPrems
- ( ) Infloran
- ( ) Labinic
- ( ) Other: ____________________

6. What gestational age group receive probiotics? *

____

7. Does birthweight influence probiotic use? *

Mark only one oval.

- ( ) Yes
- ( ) No

8. If birthweight does influence probiotic use, please explain:

____
____
____
____
____

9. Does your unit have any circumstances in which probiotic administration is contraindicated e.g. congenital bowel atresia? *

Mark only one oval.

- Yes
- No
- Other: ______________________

10. When does your unit start probiotics? *

Mark only one oval.

- At birth
- After commencing enteral feeds
- Once the baby reaches full enteral feeds
- Other: ______________________

11. At what point would your unit discontinue probiotic supplementation e.g. at how many weeks postmenstrual age (PMA)? *

____

12. Does your unit have a probiotics guideline? *

Mark only one oval.

- Yes
- No
- Don't know

13. Is your Microbiology lab aware of your unit using probiotics? *

Mark only one oval.

- Yes
- No
- Don't know

14. If a baby is on IV antibiotics for suspected sepsis, is your unit policy to: *

Mark only one oval.

- Stop probiotics completely
- Reduce/modify the dosage
- Continue with probiotics

15. If a baby's enteral feeds are withheld, does your unit: *

Mark only one oval.

- Stop probiotic supplementation whilst feeds are withheld
- Reduce/modify the dosage of the probiotics whilst feeds are withheld
- Continue probiotics whilst feeds are withheld

16. If you are treating a baby for necrotising enterocolitis, does your unit: *

Mark only one oval.

- Stop probiotics supplementation whilst feeds are withheld
- Reduce/modify the dosage of the probiotics whilst feeds are withheld
- Continue probiotics whilst feeds are withheld

17. Following treatment of necrotising enterocolitis, when are probiotics restarted? *

Mark only one oval.

- When antibiotics are completed
- When enteral feeds are restarted
- When the baby reaches full enteral feeds
- Never
- Other: ______________________

18. Does your unit provide a probiotic parent information leaflet? *

Mark only one oval.

- Yes
- No
- Don't know

19. Has your unit experienced any parental refusal for probiotics? *

Mark only one oval.

- Yes
- No

Neonatal units not utilising probiotics

20. Have you encountered parents enquiring about probiotics? *

Mark only one oval.

- Yes
- No

21. Is your unit planning to use probiotics within the next 12 months? *

Mark only one oval.

- Yes
- No

This content is neither created nor endorsed by Google.

Google Forms
