# Supplementary material for: How frequent is routine use of probiotics in UK neonatal units?
Source: BMJ Paediatr Open. 2023 Jul 14;7(1):e002012. doi: 10.1136/bmjpo-2023-002012 (PMC10351264; doi:10.1136/bmjpo-2023-002012)
Supplement: Author's manuscript [file bmjpo-2023-002012.draft_revisions.pdf]

BMJ Paediatrics Open

BMJ Paediatrics Open is committed to open peer review. As part of this commitment we make the peer review history of every article we publish publicly available.

When an article is published we post the peer reviewers' comments and the authors' responses online. We also post the versions of the paper that were used during peer review. These are the versions that the peer review comments apply to.

The versions of the paper that follow are the versions that were submitted during the peer review process. They are not the versions of record or the final published versions. They should not be cited or distributed as the published version of this manuscript.

BMJ Paediatrics Open is an open access journal and the full, final, typeset and author-corrected version of record of the manuscript is available on our site with no access controls, subscription charges or pay-per-view fees (http://bmjpaedsopen.bmj.com).

If you have any questions on BMJ Paediatrics Open's open peer review process please email info.bmjpo@bmj.com

BMJ Paediatrics Open

How Frequent is Routine Use of Probiotics in UK Neonatal Units?

Journal:	*BMJ Paediatrics Open*
Manuscript ID	bmjpo-2023-002012
Article Type:	Original research
Date Submitted by the Author:	06-Apr-2023
Complete List of Authors:	Patel, Neaha; Homerton Healthcare NHS Foundation Trust, Department of Neonatology<br>Collaborative Group, NeoTRIPS; Chelsea and Westminster Hospital NHS Foundation Trust<br>Evans, Katie; Imperial College London, Department of Medicine, Neonatal Data Analysis Unit, Section of Neonatal Medicine, Chelsea and Westminster campus<br>Berrington, Janet; Newcastle Upon Tyne Hospitals NHS Foundation Trust, Department of Neonatology; Newcastle University<br>Szatkowski, Lisa; Newcastle Upon Tyne Hospitals NHS Foundation Trust, Department of Neonatology; Newcastle University<br>Costeloe, Kate; Homerton Healthcare NHS Foundation Trust, Department of Neonatology; Queen Mary University of London Barts and The London School of Medicine and Dentistry, Genomics and Child Health<br>Ojha, Shalini; University of Nottingham Faculty of Medicine and Health Sciences, Centre for Perinatal Research, Lifespan and Population Health, School of Medicine<br>Fleming, PF; Homerton University Hospital NHS Foundation Trust, Department of Neonatology; Queen Mary University of London Barts and The London School of Medicine and Dentistry, Genomics and Child Health<br>Battersby, Cheryl; Imperial College London, Department of Medicine, Neonatal Data Analysis Unit, Section of Neonatal Medicine, Chelsea and Westminster campus
Keywords:	Neonatology, Microbiology

SCHOLARONE™
Manuscripts

I, the Submitting Author has the right to grant and does grant on behalf of all authors of the Work (as defined in the below author licence), an exclusive licence and/or a non-exclusive licence for contributions from authors who are: i) UK Crown employees; ii) where BMJ has agreed a CC-BY licence shall apply, and/or iii) in accordance with the terms applicable for US Federal Government officers or employees acting as part of their official duties; on a worldwide, perpetual, irrevocable, royalty-free basis to BMJ Publishing Group Ltd ("BMJ") its licensees and where the relevant Journal is co-owned by BMJ to the co-owners of the Journal, to publish the Work in this journal and any other BMJ products and to exploit all rights, as set out in our licence.

The Submitting Author accepts and understands that any supply made under these terms is made by BMJ to the Submitting Author unless you are acting as an employee on behalf of your employer or a postgraduate student of an affiliated institution which is paying any applicable article publishing charge ("APC") for Open Access articles. Where the Submitting Author wishes to make the Work available on an Open Access basis (and intends to pay the relevant APC), the terms of reuse of such Open Access shall be governed by a Creative Commons licence – details of these licences and which Creative Commons licence will apply to this Work are set out in our licence referred to above.

Other than as permitted in any relevant BMJ Author's Self Archiving Policies, I confirm this Work has not been accepted for publication elsewhere, is not being considered for publication elsewhere and does not duplicate material already published. I confirm all authors consent to publication of this Work and authorise the granting of this licence.

Confidential: For Review Only

How Frequent is Routine Use of Probiotics in UK Neonatal Units?

Neaha Patel[1], NeoTRIPS Collaborative Group*; Katie Evans[3], Janet Berrington[4], Lisa Szatkowski[4], Kate Costeloe[1, 2], Shalini Ojha[5], †Paul Fleming[1, 2] & †Cheryl Battersby[3]

Affiliations

1. Department of Neonatology, Homerton Healthcare NHS Foundation Trust, London, United Kingdom
2. Genomics and Child Health, Barts and The London School of Medicine and Dentistry, QMUL, London, United Kingdom
3. Department of Medicine, Neonatal Data Analysis Unit, Section of Neonatal Medicine, Chelsea and Westminster campus, Imperial College London, London, United Kingdom
4. Department of Neonatology, Newcastle Upon Tyne Foundation Hospitals Trust, Newcastle, UK and Newcastle University, Newcastle, United Kingdom
5. Centre for Perinatal Research, Lifespan and Population Health, School of Medicine, University of Nottingham, United Kingdom

* Hosted by Chelsea and Westminster Hospital NHS Foundation Trust, London, United Kingdom

†Denotes equal author contribution

Confidential: For Review Only

ABSTRACT

Objective: There is a lack of UK guidance regarding routine use of probiotics in preterm infants to prevent necrotising enterocolitis, late-onset sepsis, and death. As practices can vary, we aimed to determine the current usage of probiotics within neonatal units in the United Kingdom.

Design and setting: Using NeoTRIPS, a trainee-led neonatal research network, an online survey was disseminated to neonatal units of all service levels within England, Scotland, Northern Ireland, and Wales in 2022. Trainees were requested to complete one survey per unit regarding routine probiotic administration.

Results: 161/188 (86%) neonatal units responded to the survey. 70/161 (44%) respondents routinely give probiotics to preterm infants. 45/70 (64%) use the probiotic product *Lactobacillus acidophilus* NCFM/*Bifidobacterium bifidum* Bb-06/*Bifidobacterium infantis* Bi-26 (Labinic™). 57/70 (81%) start probiotics in infants ≤32 weeks gestation. 33/70 (47%) had microbiology departments that were aware of the use of probiotics and 64/70 (91%) had a guideline available. Commencing enteral feeds was a prerequisite to starting probiotics in 62/70 (89%) units. The majority would stop probiotics if enteral feeds were withheld (59/70; 84%) or if the infant was being treated for necrotising enterocolitis (69/70; 99%). 24/91 (26%) units that did not use probiotics at the time of the survey were planning to introduce them within the next 12 months.

Conclusions: More than 40% of all UK neonatal units that responded are now routinely administering probiotics, with variability in the product used. With increased probiotic

Confidential: For Review Only

usage in recent years, there is a need to establish whether this translates to improved clinical outcomes.

What is already known on this topic?

- A 2018 survey of probiotic use in the UK reported that 17% of tertiary-level neonatal intensive care units were routinely using probiotics.
- There are inconsistencies in the body of literature as to whether probiotics can prevent necrotising enterocolitis, late-onset sepsis and death.
- Many trials evaluating probiotics in preterm infants have used different strains, contributing to lack of consensus on which product to use.

What this study adds?

- Over 40% of responding UK neonatal units and 62% of responding tertiary-level neonatal intensive care units currently use probiotics.
- There is heterogeneity in the probiotic product used.
- The increase in use offers the opportunity for further national research reviewing the impact of probiotics use on clinical outcomes.

How this study might affect research, practice or policy?

- This survey may inform future studies that evaluate efficacy of routine probiotic administration.

Confidential: For Review Only

INTRODUCTION

Routine administration of probiotics to prevent necrotising enterocolitis (NEC), late-onset sepsis (LOS) and death in preterm infants remains contentious. Evidence of benefit from large randomised controlled trials (RCTs), together with data from pre- and post-implementation studies is inconsistent.[1-4] The most recent Cochrane meta-analysis stated that probiotics *may* reduce the risk of NEC [RR 0.54, 95% CI 0.45 to 0.65 (54 trials, 10,604 infants; I^2 = 17%)] and *probably* reduce mortality [RR 0.76, 95% CI 0.65 to 0.89; (51 trials, 10,170 infants; I^2 = 0%)] and late-onset invasive infection [RR 0.89, 95% CI 0.82 to 0.97; (47 trials, 9762 infants; I^2 = 19%)]. This review showed limited evidence of benefit for infants <1000g and recommended further assessment of probiotics in RCTs so long as families and caregivers supported such a study.[5]

Historical uncertainties around optimal probiotic strains for use in preterm infants and of probiotics safety has likely contributed to a lower uptake of their use.[6] A large network meta-analysis comprehensively evaluated efficacy of different probiotic strains and found that some may have more evidence of benefit than others.[7] The same review cautions that without clear evidence of efficacy for some probiotics, *'clinicians may be left using inadequately tested, potentially unsafe and possibly ineffective treatments'*. A 2018 telephone survey (with a 100% response rate) of 58 neonatal intensive care units providing tertiary-level care in the United Kingdom (UK), revealed that 17% of centres routinely administered probiotics.[8] In 2020, The European Society for Paediatric Gastroenterology Hepatology and Nutrition

https://mc.manuscriptcentral.com/bmjpo

(ESPGHAN) made conditional recommendations for certain probiotic strains in preterm infants but cautioned that these were made with low certainty of benefit.[9] Parent-advocacy groups support probiotic use.[10]

In light of the most recent Cochrane review and ESPGHAN recommendations, it is likely that more UK neonatal units are moving to routine probiotic administration despite uncertainties around evidence of benefit. In this study, we aimed to identify current practice on probiotic use in UK neonatal units using the Neonatal Trainee Led Research and Improvement Projects (NeoTRIPS) network, a UK national neonatal trainee-led research initiative.

Confidential: For Review Only

METHODS

A 21-item exploratory survey instrument (online supplementary material) was developed by the research team in May 2022 to determine the current use of probiotics in UK neonatal units. The structure was designed in '*Google Forms*'[11] in which responders were asked three questions to acquire basic neonatal unit information, including the neonatal Operational Delivery Network (ODN) location and unit care level (tick-box responses) and the name of the neonatal unit (free text response). After this, based on a *yes/no* answer to the following screening question 'does your neonatal unit currently use probiotics?', participants were directed to one of two different sets of questions.

Participants responding *'no'* to the initial screening question were directed to two further multiple-choice questions asking if they intended to implement routine probiotic use within the next 12 months and whether parents had made enquiries about probiotics. *'Yes'* responders were steered to 15 additional questions to ascertain unit probiotic administration practices. These consisted of 12 multiple-choice style questions, with four containing an optional free text response, and three open-ended questions. These questions captured the following information: which probiotic product was being used; probiotic initiation criteria (based on gestational age, birthweight, and enteral feeds); postmenstrual gestation at which probiotics are discontinued; contraindications to probiotic use; presence of a unit-specific probiotics guideline and parent information leaflets; microbiology department awareness of neonatal probiotic

https://mc.manuscriptcentral.com/bmjpo

use; and probiotic administration during scenarios when an infant had sepsis, NEC and/or was made nil by mouth. The survey was trialled by the research team which comprised a medical statistician, five neonatal consultants and two senior neonatal trainees and underwent five iterations before the content was finalised. The research team aimed for a minimum 70% response rate to the survey based upon similar targets set for other UK neonatal clinical practice surveys.[12]

The survey was subsequently disseminated by the NeoTRIPS collaborative group. NeoTRIPS is a national, trainee-led, neonatal research network.[13] Regional NeoTRIPS leads for each neonatal ODN were provided with the survey link to disseminate to local NeoTRIPS leads for each neonatal unit. The survey invitation contained instructions for one survey to be completed per unit by a trainee. Survey reminders were sent to the regional NeoTRIPS leads fortnightly during the survey period.

There are 15 neonatal ODNs including 188 units in the UK. Centres caring for newborn infants in the UK are stratified into three levels: level 1 which refers to special care units (SCUs); level 2 or local neonatal units (LNUs); and highly specialised level 3 neonatal intensive care units (NICUs).[14]

The survey results were collated in a Microsoft Excel file and data were summarised using descriptive statistics for quantitative responses.

https://mc.manuscriptcentral.com/bmjpo

Confidential: For Review Only

Patient and Public Involvement

In August 2022, focus group sessions with parents, former NICU patients and advocacy groups recruited through the neoWONDER collaborative [15] were held to establish perspectives on the use of routinely collected neonatal data to monitor probiotic efficacy during routine administration. It was explained to participants that the survey report could be instrumental in designing any future studies and the members of this group felt this was important. The focus group was not involved in the design of this survey of clinicians' clinical practice.

Confidential: For Review Only

RESULTS

Survey response rate and probiotic use

Between 13th August 2022 and 31st October 2022, 161/188 (86%) neonatal units completed the survey. Figure 1 outlines the response rate and probiotic use by unit designation.

Survey responses for units routinely using probiotics

Table 1 outlines survey responses from the 70 units that confirmed routine probiotic use.

Confidential: For Review Only

Table 1: Survey results for units using probiotics (*n*=70)	
**Survey question**	***n* (%)**
**Probiotic choice**	
Lactobacillus acidophilus NCFM/*Bifidobacterium bifidum* Bb-06/*Bifidobacterium* infantis Bi-26 (Labinic™)	45 (64)
Bifidobacterium infantis Bb-02/*Bifidobacterium lactis* Bb-12/*Streptococcus thermophilus* (ProPrems®)	15 (21)
Bifidobacterium bifidum/Lactobacillus acidophilus (Infloran®)	10 (14)
**Gestation cut-off for probiotic administration**	
≤34 weeks	10 (14)
≤33 weeks	2 (3)
≤32 weeks	57 (81)
≤28 weeks	1 (1)
**When probiotics are started**	
After enteral feeds are commenced	62 (89)
Soon after birth	8 (11)
**Guideline for probiotic administration**	
Yes	64 (91)
No	4 (6)
Unknown	2 (3)
**Microbiology team awareness of neonatal probiotic use**	
Yes	33 (47)
No	2 (3)
Unknown	35 (50)
**Probiotic use during antibiotic administration**	
Continue	55 (79)
Stop	15 (21)
Reduce or modify	0 (0)
**Parent information leaflets**	
Yes	32 (46)
No	30 (43)
Unknown	8 (11)
**Parental refusal of probiotics**	
Yes	5 (7)
No	65 (93)

view Only

Additional responses from units using probiotics

Birthweight:

59/70 (84%) units using probiotics additionally had birthweight as part of their probiotic administration criteria, with 56/59 (95%) starting probiotics in infants ≤1500g; 1/59 (2%) in infants ≤1600g; 1/59 (2%) in infants ≤1800g; and 1/59 (2%) not specifying a birthweight criterion.

Confidential: For Review Only

When to stop probiotics:

The majority of units [55/70 (79%)] would discontinue probiotics at 34 weeks postmenstrual age, with 1/70 (1%) discontinuing at 32 weeks; 2/70 (3%) at 35 weeks; 2/70 (3%) at 36 weeks; and 2/70 (3%) at term. 3/70 (4%) units would stop probiotics at discharge regardless of postmenstrual age. 5/70 (7%) units would discontinue probiotics at either 34 weeks postmenstrual age or discharge, depending on whether they were commenced due to gestational age or birthweight, respectively.

Contraindications to using probiotics:

31/70 (44%) units listed contraindications to starting probiotics, and where free text comments were provided, these included: intestinal atresia; NEC; sepsis and/or suspected immunocompromise.

Probiotic use during abdominal concerns including necrotising enterocolitis

If enteral feeds are stopped, 59/70 (84%) units would also stop probiotics; 9/70 (13%) would continue and 2/70 (3%) would reduce or modify the dose.

69/70 (99%) units would stop probiotics completely for infants being treated for NEC, and just one unit stated that they would continue. Probiotics would be restarted after an episode of NEC once enteral feeds are reintroduced and/or a set volume of feed is tolerated in 53/69 (77%) units. In 11/69 (16%) units, they would only be restarted based on a consultant and/or surgical team decision. One unit stated they would not restart probiotics after NEC, one would only restart once antibiotics (for NEC) are stopped, and one would only restart once the baby was repatriated to their local hospital from the surgical centre. 2/69 (3%) units were unsure regarding their policy of restarting probiotics after an episode of NEC.

Confidential: For Review Only

<u>Survey responses for units not using probiotics</u>

Table 2 outlines survey responses from the 91 units that do not routinely use probiotics.

Table 2: Survey results for units not using probiotics (*n*=91)

Survey question	*n* (%)
**Intention to introduce routine probiotics within the next 12 months**	24 (26)
Yes	67 (74)
No	
**Parents enquiring regarding probiotic use**	
Yes	18 (20)
No	73 (80)

DISCUSSION

This up-to-date survey with a high response rate represents national practice relating to probiotic use in UK neonatal units. The survey confirms a notable increase in routine probiotic administration, especially in NICUs (now 62%), compared to the last survey in 2018 when this figure was 17%.[8] This may be due to the recent position paper from ESPGHAN recommending specific probiotic strains to reduce rates of NEC (despite low certainty of benefit), which may in turn have increased clinician confidence in using probiotic interventions.[9]

The most frequently used probiotic product in UK neonatal units at the time the survey was conducted was a combination of *Lactobacillus acidophilus*, *Bifidobacterium bifidum* Bb-06 and *Bifidobacterium infantis* Bi-26 (Labinic™). This formulation has not been extensively evaluated in large RCTs[16] and does not meet current ESPGHAN guidelines but has been reported to show efficacy against NEC, LOS and unadjusted mortality in one study[3]. The second most commonly used combination is *Bifidobacterium infantis*, *Bifidobacterium lactis* Bb-12 and *Streptococcus thermophiles* (ProPrems®). This latter combination of probiotic bacteria has been evaluated in two RCTs, but neither was powered for NEC as a primary outcome.[1,17]

Choosing which probiotic to use on a neonatal unit can sometimes be dependent upon availability rather than using products which have robust evidence of benefit. Van den Akker *et al* cautioned against this, stating that *'clinicians may be left using inadequately*

https://mc.manuscriptcentral.com/bmjpo

Confidential: For Review Only

tested, potentially unsafe and possibly ineffective treatments'.[7] Furthermore, there is inconsistency in the evidence of benefit in pre- and post-implementation studies using Labinic™ and the combination of *Bifidobacterium bifidum* and *Lactobacillus acidophilus* (Infloran®) in UK neonatal units. Whilst one study reported a significant NEC reduction during epochs after these probiotics were routinely implemented,[3] another more recent study did not show evidence of benefit.[4] Why these differences exist in different geographic locations but within the same country is unclear, but may reflect differences in infants, unit practices or disease categorisation.

Inconsistencies in probiotic efficacy are also recognised across large RCTs. Among the two largest, the ProPrems trial reported a significant reduction in NEC amongst infants randomised to a probiotic combination containing *Bifidobacterium infantis, Streptococcus thermophilus* and *Bifidobacterium lactis*;[1] however, for participants in the PiPS trial, there was no evidence of NEC reduction among infants randomised to *Bifidobacterium breve* BBG-001.[2] Neither trial reported significant reductions in LOS or mortality. Strain selection and probiotic combinations are therefore important and may act differently in different populations. Not all are effective, and none has been shown to definitively reduce all of NEC, LOS and death.

Probiotic safety is frequently highlighted when considering routine probiotic supplementation in preterm infants. Adverse clinical events have mostly taken the form of isolated case reports of bacteraemias and given the large numbers of preterm babies exposed to probiotics in clinical trials, these reports appear less frequently.[18]

Confidential: For Review Only

Probiotic contamination has also been reported and associated with death from fungal contaminants.[19] Using probiotic products that are manufactured to the highest pharmaceutical standard is thus imperative. In this current survey, more than 50% of respondents were unsure if their local microbiology department was aware that probiotics were being used on the neonatal unit. Whilst this may represent the responses of more junior members of the team, it also potentially highlights the lack of appreciation/awareness of the importance of involving local laboratory services to ensure there is a system in place to detect adverse events associated with probiotic use. When using live interventions, it is imperative that unit guidelines are designed with input from local microbiology teams so that patient and product safety can be monitored.[20]

Amongst units not currently using probiotics, 26% (24 units) indicated they intend to start routine probiotic administration within the next year. If this occurs, it will see half of all UK neonatal units using probiotics. With the increasing number of units moving towards routine administration, it is important that probiotic efficacy is monitored systematically across the UK's neonatal population. One way this could be achieved is to monitor efficacy using large population databases of routinely-collected electronic patient record data, such as the National Neonatal Research Database (NNRD). To do this, these databases would need to record probiotic use in a manner that allows easy extraction of these data and reliable comparisons of clinical endpoints in babies exposed to probiotics versus those who have not been exposed. During patient and parent focus groups associated with designing this project, participants were

Confidential: For Review Only

unanimously supportive towards maximising the utility of routinely collected data to address some of the uncertainties around probiotic efficacy. Monitoring safety during routine implementation is equally important and utilising systems such as the British Paediatric Surveillance Unit could be one way to capture and report any adverse events associated with probiotic use.[21]

One of the greatest strengths of this study was the high response rate of 86%. This exceeded the target of 70% and demonstrates the willingness of trainees to undertake research. Furthermore, the survey was completed within 12 weeks making results available in a timely and efficient manner. The main limitation of the study was that parts of the survey that allowed free text entries were not always completed (e.g., contraindications to using probiotics).

Confidential: For Review Only

CONCLUSIONS

This survey confirms that an increasing number of UK neonatal units are administering probiotics to preterm babies. As units move towards routine probiotic use, systems to monitor efficacy are needed as some of the probiotics being used have limited evidence of benefit. Evaluation of efficacy could be achieved by interrogating routinely collected national datasets. Robust reporting of any adverse outcomes associated with probiotic use are also needed so that adequate safety monitoring occurs in parallel with routine implementation.

Acknowledgements

We would like to thank all the neonatal units that contributed to this survey.

Contributors

NP, KE, JB, LS, KC, SO, PF and CB were involved in the conception, methodology, writing, reviewing, and editing of the manuscript. NP, KE, and the NeoTRIPS Collaborative Group were involved in data acquisition.

Funding

No formal funding was provided for this study.

Competing Interests

None of the authors has a conflict of interest to declare.

Confidential: For Review Only

Patient consent for publication

Not required.

Ethical approval

Formal ethical approval was not required for this evaluation of service provision. No patient identifiable information was sought or gathered in this survey.

Data availability statement

All data relevant to the study are included in the article or uploaded as supplementary information.

Confidential: For Review Only

REFERENCES

1. Jacobs SE, Tobin JM, Opie GF, *et al*. Probiotic effects on late-onset sepsis in very preterm infants: a randomized controlled trial. *Pediatrics* 2013;132(6):1055-62. doi: 10.1542/peds.2013-1339 [published Online First: 20131118]
2. Costeloe K, Bowler U, Brocklehurst P, *et al*. A randomised controlled trial of the probiotic Bifidobacterium breve BBG-001 in preterm babies to prevent sepsis, necrotising enterocolitis and death: the Probiotics in Preterm InfantS (PiPS) trial. *Health Technol Assess* 2016;20(66):1-194. doi: 10.3310/hta20660
3. Robertson C, Savva GM, Clapuci R, *et al*. Incidence of necrotising enterocolitis before and after introducing routine prophylactic Lactobacillus and Bifidobacterium probiotics. *Arch Dis Child Fetal Neonatal Ed* 2020;105(4):380-86. doi: 10.1136/archdischild-2019-317346 [published Online First:20191030]
4. Granger C, Dermyshi E, Roberts E, *et al*. Necrotising enterocolitis, late-onset sepsis and mortality after routine probiotic introduction in the UK. *Arch Dis Child Fetal Neonatal Ed* 2022;107(4):352-58. doi: 10.1136/archdischild-2021-322252 [published Online First: 20210825]
5. Sharif S, Meader N, Oddie SJ, *et al*. Probiotics to prevent necrotising enterocolitis in very preterm or very low birth weight infants. *Cochrane Database Syst Rev* 2020;10(10):CD005496. doi: 10.1002/14651858.CD005496.pub5 [published Online First: 20201015]

Confidential: For Review Only

6. Jarrett P, Meczner A, Costeloe K, *et al*. Historical aspects of probiotic use to prevent necrotising enterocolitis in preterm babies. *Early Hum Dev* 2019;135:51-57. doi: 10.1016/j.earlhumdev.2019.05.015 [published Online First: 20190529]

7. van den Akker CHP, van Goudoever JB, Szajewska H, *et al*. Probiotics for Preterm Infants: A Strain-Specific Systematic Review and Network Meta-analysis. *J Pediatr Gastroenterol Nutr* 2018;67(1):103-22. doi:10.1097/MPG.0000000000001897

8. Duffield SD, Clarke P. Current use of probiotics to prevent necrotising enterocolitis. *Arch Dis Child Fetal Neonatal Ed* 2019;104(2):F228. doi: 10.1136/archdischild-2018-316199 [published Online First: 20181121]

9. Van den Akker CHP, van Goudoever JB, Shamir R, *et al*. Probiotics and Preterm Infants: A Position Paper by the European Society for Paediatric Gastroenterology Hepatology and Nutrition Committee on Nutrition and the European Society for Paediatric Gastroenterology Hepatology and Nutrition Working Group for Probiotics and Prebiotics. *J Pediatr Gastroenterol Nutr* 2020;70(5):664-80. doi: 10.1097/MPG.0000000000002655

10. NEC UK. NEC UK [online]. 2018. www.necuk.org.uk (accessed 4th April 2023)

11. Google. Google Forms [online]. https://docs.google.com/forms (accessed 4th April 2023)

12. Dorling J, Tume L, Arch B, *et al*. Gastric residual volume measurement in British neonatal intensive care units: a survey of practice. *BMJ Paediatr Open* 2020;4(1):e000601. doi: 10.1136/bmjpo-2019-000601 [published Online First: 20200807]

Confidential: For Review Only

https://mc.manuscriptcentral.com/bmjpo

13. NeoTRIPS. NeoTRIPS [online]. 2022. https://neotrips.org (accessed 4th April 2023)

14. Department of Health. Toolkit for high quality neonatal services [online]. 2009. https://www.londonneonatalnetwork.org.uk/wp-content/uploads/2015/09/Toolkit-2009.pdf (accessed 4th April 2023)

15. neoWONDER. neoWONDER [online]. 2022. https://www.neowonder.org.uk/ (accessed 4th April 2023)

16. Sowden M, van Niekerk E, Bulabula ANH, *et al*. Effect of a Multi-Strain Probiotic on Growth and Time to Reach Full Feeds in Preterm Neonates. *Nutrients* 2022;14(21) doi: 10.3390/nu14214658 [published Online First: 20221103]

17. Bin-Nun A, Bromiker R, Wilschanski M, *et al*. Oral probiotics prevent necrotizing enterocolitis in very low birth weight neonates. *J Pediatr* 2005;147(2):192-6. doi: 10.1016/j.jpeds.2005.03.054

18. Fleming PF, Berrington JE, Jacobs SE. Addressing safety concerns of probiotic use in preterm babies. *Early Hum Dev* 2019;135:72-74. doi: 10.1016/j.earlhumdev.2019.05.016 [published Online First: 20190530]

19. Vallabhaneni S, Walker TA, Lockhart SR, *et al*. Notes from the field: Fatal gastrointestinal mucormycosis in a premature infant associated with a contaminated dietary supplement--Connecticut, 2014. *MMWR Morb Mortal Wkly Rep* 2015;64(6):155-6.

20. Barbian ME, Buckle R, Denning PW, *et al*. To start or not: Factors to consider when implementing routine probiotic use in the NICU. *Early Hum Dev*

Confidential: For Review Only

2019;135:66-71. doi: 10.1016/j.earlhumdev.2019.05.009 [published Online First: 20190610]

21. British Paediatric Surveillance Unit. Surveillance of Rare Childhood Disease [online]. 2019. https://bpsu.org.uk (accessed 4th April 2023)

Confidential: For Review Only

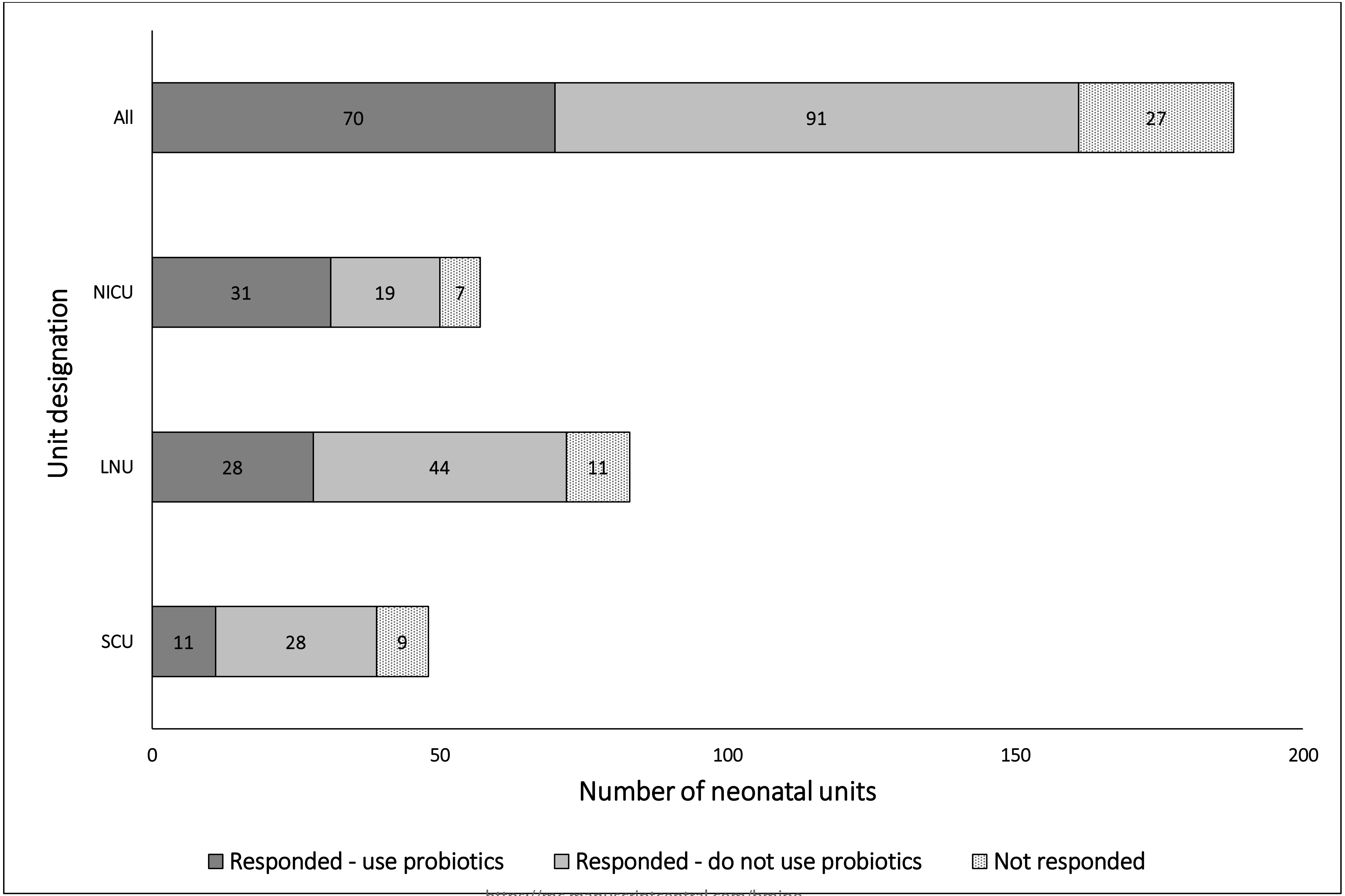
Unit designation
All
NICU
LNU
SCU
70
91
27
31
19
7
28
44
11
11
28
9
0
50
100
150
200
Number of neonatal units
Responded - use probiotics
Responded - do not use probiotics
Not responded

Evaluating the use of probiotics in UK neonatal units: a national, trainee-led survey

This is a national, trainee-led survey evaluating the use of probiotics in all UK neonatal units, conducted as part of NeoTRIPS.

NeoTRIPS is a national neonatal study group, using the power of trainees to improve neonatal care through large scale quality improvement and research projects.

Please ensure that only 1 trainee responds per neonatal unit.

* Required

1. What is the name of your neonatal operational delivery network (ODN)? *

   Mark only one oval.

   - East Midlands Neonatal ODN
   - East of England Neonatal ODN
   - Neonatal Network Northern Ireland
   - North Central and East London Neonatal Network
   - North West London Neonatal ODN
   - North West Neonatal ODN
   - Northern Neonatal Network
   - Scottish Perinatal Network
   - South East Coast Neonatal Network
   - South London Network
   - South West Neonatal Network
   - Thames Valley & Wessex Neonatal Network
   - Wales Maternity and Neonatal Network
   - West Midlands Neonatal ODN
   - Yorkshire & Humber Neonatal ODN

2. What is the name of your neonatal unit/local hospital? *

   ____

3. What is the level of your neonatal unit? *

   Mark only one oval.

   - Level 1
   - Level 2
   - Level 3

4. Does your neonatal unit currently use probiotics? *

Mark only one oval.

- Yes *Skip to question 5*
- No *Skip to question 20*
- Don't know

Neonatal units utilising probiotics

5. What probiotic do you currently use? *

Mark only one oval.

- ProPrems
- Infloran
- Labinic
- Other: ____________________

6. What gestational age group receive probiotics? *

____

7. Does birthweight influence probiotic use? *

Mark only one oval.

- Yes
- No

8. If birthweight does influence probiotic use, please explain:

____
____
____
____
____

9. Does your unit have any circumstances in which probiotic administration is contraindicated e.g. congenital bowel atresia? *

Mark only one oval.

- Yes
- No
- Other: ____________________

10. When does your unit start probiotics? *

Mark only one oval.

- At birth
- After commencing enteral feeds
- Once the baby reaches full enteral feeds
- Other: ____________________

11. At what point would your unit discontinue probiotic supplementation e.g. at how many weeks postmenstrual age (PMA)? *

____

12. Does your unit have a probiotics guideline? *

Mark only one oval.

- Yes
- No
- Don't know

13. Is your Microbiology lab aware of your unit using probiotics? *

Mark only one oval.

- Yes
- No
- Don't know

14. If a baby is on IV antibiotics for suspected sepsis, is your unit policy to: *

Mark only one oval.

- Stop probiotics completely
- Reduce/modify the dosage
- Continue with probiotics

15. If a baby's enteral feeds are withheld, does your unit: *

Mark only one oval.

- Stop probiotic supplementation whilst feeds are withheld
- Reduce/modify the dosage of the probiotics whilst feeds are withheld
- Continue probiotics whilst feeds are withheld

16. If you are treating a baby for necrotising enterocolitis, does your unit: *

Mark only one oval.

- Stop probiotics supplementation whilst feeds are withheld
- Reduce/modify the dosage of the probiotics whilst feeds are withheld
- Continue probiotics whilst feeds are withheld

17. Following treatment of necrotising enterocolitis, when are probiotics restarted? *

Mark only one oval.

- When antibiotics are completed
- When enteral feeds are restarted
- When the baby reaches full enteral feeds
- Never
- Other: ____________________

18. Does your unit provide a probiotic parent information leaflet? *

Mark only one oval.

- Yes
- No
- Don't know

19. Has your unit experienced any parental refusal for probiotics? *

Mark only one oval.

- Yes
- No

Neonatal units not utilising probiotics

20. Have you encountered parents enquiring about probiotics? *

Mark only one oval.

- Yes
- No

21. Is your unit planning to use probiotics within the next 12 months? *

Mark only one oval.

- Yes
- No

This content is neither created nor endorsed by Google.

Google Forms

BMJ Paediatrics Open

How Frequent is Routine Use of Probiotics in UK Neonatal Units?

Journal:	*BMJ Paediatrics Open*
Manuscript ID	bmjpo-2023-002012.R1
Article Type:	Original research
Date Submitted by the Author:	26-May-2023
Complete List of Authors:	Patel, Neaha; Homerton Healthcare NHS Foundation Trust, Department of Neonatology<br>Collaborative Group, NeoTRIPS; Chelsea and Westminster Hospital NHS Foundation Trust<br>Evans, Katie; Imperial College London, Neonatal Medicine, School of Public Health Faculty of Medicine, Chelsea and Westminster campus<br>Berrington, Janet; Newcastle Upon Tyne Hospitals NHS Foundation Trust, Department of Neonatology; Newcastle University, Translational and Clinical Research Institute<br>Szatkowski, Lisa; University of Nottingham Faculty of Medicine and Health Sciences, Centre for Perinatal Research, Lifespan and Population Health, School of Medicine<br>Costeloe, Kate; Homerton Healthcare NHS Foundation Trust, Department of Neonatology; Queen Mary University of London Barts and The London School of Medicine and Dentistry, Genomics and Child Health<br>Ojha, Shalini; University of Nottingham Faculty of Medicine and Health Sciences, Centre for Perinatal Research, Lifespan and Population Health, School of Medicine; University Hospitals of Derby and Burton NHS Foundation Trust, Neonatal Unit<br>Fleming, Paul; Homerton University Hospital NHS Foundation Trust, Department of Neonatology; Queen Mary University of London Barts and The London School of Medicine and Dentistry, Genomics and Child Health<br>Battersby, Cheryl; Imperial College London, Neonatal Medicine, School of Public Health Faculty of Medicine, Chelsea and Westminster campus
Keywords:	Neonatology, Microbiology

SCHOLARONE™
Manuscripts

I, the Submitting Author has the right to grant and does grant on behalf of all authors of the Work (as defined in the below author licence), an exclusive licence and/or a non-exclusive licence for contributions from authors who are: i) UK Crown employees; ii) where BMJ has agreed a CC-BY licence shall apply, and/or iii) in accordance with the terms applicable for US Federal Government officers or employees acting as part of their official duties; on a worldwide, perpetual, irrevocable, royalty-free basis to BMJ Publishing Group Ltd ("BMJ") its licensees and where the relevant Journal is co-owned by BMJ to the co-owners of the Journal, to publish the Work in this journal and any other BMJ products and to exploit all rights, as set out in our licence.

The Submitting Author accepts and understands that any supply made under these terms is made by BMJ to the Submitting Author unless you are acting as an employee on behalf of your employer or a postgraduate student of an affiliated institution which is paying any applicable article publishing charge ("APC") for Open Access articles. Where the Submitting Author wishes to make the Work available on an Open Access basis (and intends to pay the relevant APC), the terms of reuse of such Open Access shall be governed by a Creative Commons licence – details of these licences and which Creative Commons licence will apply to this Work are set out in our licence referred to above.

Other than as permitted in any relevant BMJ Author's Self Archiving Policies, I confirm this Work has not been accepted for publication elsewhere, is not being considered for publication elsewhere and does not duplicate material already published. I confirm all authors consent to publication of this Work and authorise the granting of this licence.

Confidential: For Review Only

How Frequent is Routine Use of Probiotics in UK Neonatal Units?

Neaha Patel [1], [+]NeoTRIPS Collaborative Group; Katie Evans [3], Janet Berrington [4], Lisa Szatkowski [5], Kate Costeloe [1, 2], Shalini Ojha [5, 6], [*]Paul Fleming [1, 2] & [*]Cheryl Battersby [3]

Affiliations

1. Department of Neonatology, Homerton Healthcare NHS Foundation Trust, London, United Kingdom
2. Genomics and Child Health, Barts and The London School of Medicine and Dentistry, QMUL, London, United Kingdom
3. Neonatal Medicine, School of Public Health Faculty of Medicine, Chelsea and Westminster campus, Imperial College London, London, United Kingdom
4. Department of Neonatology, Newcastle Upon Tyne Foundation Hospitals Trust, Newcastle, UK and Translational and Clinical Research Institute, Newcastle University, Newcastle, United Kingdom
5. Centre for Perinatal Research, Lifespan and Population Health, School of Medicine, University of Nottingham, United Kingdom
6. Neonatal Unit, University Hospitals of Derby and Burton NHS Trust, Derby, United Kingdom

[+]Supported by CW+ Charity and the Chelsea and Westminster Hospital NHS Foundation Trust, London, United Kingdom

[*]Denotes equal author contribution

Confidential: For Review Only

Confidential: For Review Only

ABSTRACT

Objective: There is a lack of UK guidance regarding routine use of probiotics in preterm infants to prevent necrotising enterocolitis, late-onset sepsis, and death. As practices can vary, we aimed to determine the current usage of probiotics within neonatal units in the United Kingdom.

Design and setting: Using NeoTRIPS, a trainee-led neonatal research network, an online survey was disseminated to neonatal units of all service levels within England, Scotland, Northern Ireland, and Wales in 2022. Trainees were requested to complete one survey per unit regarding routine probiotic administration.

Results: 161/188 (86%) neonatal units responded to the survey. 70/161 (44%) respondents routinely give probiotics to preterm infants. 45/70 (64%) use the probiotic product *Lactobacillus acidophilus* NCFM/*Bifidobacterium bifidum* Bb-06/*Bifidobacterium infantis* Bi-26 (Labinic™). 57/70 (81%) start probiotics in infants ≤32 weeks gestation. 33/70 (47%) had microbiology departments that were aware of the use of probiotics and 64/70 (91%) had a guideline available. Commencing enteral feeds was a prerequisite to starting probiotics in 62/70 (89%) units. The majority would stop probiotics if enteral feeds were withheld (59/70; 84%) or if the infant was being treated for necrotising enterocolitis (69/70; 99%). 24/91 (26%) units that did not use probiotics at the time of the survey were planning to introduce them within the next 12 months.

Conclusions: More than 40% of all UK neonatal units that responded are now routinely administering probiotics, with variability in the product used. With increased probiotic

Confidential: For Review Only

usage in recent years, there is a need to establish whether this translates to improved clinical outcomes.

What is already known on this topic?

- A 2018 survey of probiotic use in the UK reported that 17% of tertiary-level neonatal intensive care units were routinely using probiotics.
- There are inconsistencies in the body of literature as to whether probiotics can prevent necrotising enterocolitis, late-onset sepsis and death.
- Many trials evaluating probiotics in preterm infants have used different strains, contributing to lack of consensus on which product to use.

What this study adds?

- Over 40% of responding UK neonatal units and 62% of responding tertiary-level neonatal intensive care units currently use probiotics.
- There is heterogeneity in the probiotic product used.
- The increase in use offers the opportunity for further national research reviewing the impact of probiotics use on clinical outcomes.

How this study might affect research, practice or policy?

- This survey may inform future studies that evaluate efficacy of routine probiotic administration.

Confidential: For Review Only

INTRODUCTION

Routine administration of probiotics to prevent necrotising enterocolitis (NEC), late-onset sepsis (LOS) and death in preterm infants remains contentious. Evidence of benefit from large randomised controlled trials (RCTs), together with data from pre- and post-implementation studies is inconsistent.[1-4] The most recent Cochrane meta-analysis stated that probiotics *may* reduce the risk of NEC [RR 0.54, 95% CI 0.45 to 0.65 (54 trials, 10,604 infants; I^2 = 17%)] and *probably* reduce mortality [RR 0.76, 95% CI 0.65 to 0.89; (51 trials, 10,170 infants; I^2 = 0%)] and late-onset invasive infection [RR 0.89, 95% CI 0.82 to 0.97; (47 trials, 9762 infants; I^2 = 19%)]. This review showed limited evidence of benefit for infants <1000g and recommended further assessment of probiotics in RCTs so long as families and caregivers supported such a study.[5]

Historical uncertainties around optimal probiotic strains for use in preterm infants and of probiotics safety has likely contributed to a lower uptake of their use.[6] A large network meta-analysis comprehensively evaluated efficacy of different probiotic strains and found that some may have more evidence of benefit than others.[7] The same review cautions that without clear evidence of efficacy for some probiotics, *'clinicians may be left using inadequately tested, potentially unsafe and possibly ineffective treatments'*. A 2018 telephone survey (with a 100% response rate) of 58 neonatal intensive care units providing tertiary-level care in the United Kingdom (UK), revealed that 17% of centres routinely administered probiotics.[8] In 2020, The European Society for Paediatric Gastroenterology Hepatology and Nutrition

https://mc.manuscriptcentral.com/bmjpo

(ESPGHAN) made conditional recommendations for certain probiotic strains in preterm infants but cautioned that these were made with low certainty of benefit.[9] Parent-advocacy groups support probiotic use.[10]

In light of the most recent Cochrane review and ESPGHAN recommendations, it is likely that more UK neonatal units are moving to routine probiotic administration despite uncertainties around evidence of benefit. In this study, we aimed to identify current practice on probiotic use in UK neonatal units using the Neonatal Trainee Led Research and Improvement Projects (NeoTRIPS) network, a UK national neonatal trainee-led research initiative.

Confidential: For Review Only

METHODS

A 21-item exploratory survey instrument (online supplementary material) was developed by the research team in May 2022 to determine the current use of probiotics in UK neonatal units. The structure was designed in ‘*Google Forms*[11] in which responders were asked three questions to acquire basic neonatal unit information, including the neonatal Operational Delivery Network (ODN) location and unit care level (tick-box responses) and the name of the neonatal unit (free text response). After this, based on a *yes/no* answer to the following screening question ‘does your neonatal unit currently use probiotics?’, participants were directed to one of two different sets of questions.

Participants responding *‘no’* to the initial screening question were directed to two further multiple-choice questions asking if they intended to implement routine probiotic use within the next 12 months and whether parents had made enquiries about probiotics. *‘Yes’* responders were steered to 15 additional questions to ascertain unit probiotic administration practices. These consisted of 12 multiple-choice style questions, with four containing an optional free text response, and three open-ended questions. These questions captured the following information: which probiotic product was being used; probiotic initiation criteria (based on gestational age, birthweight, and enteral feeds); postmenstrual gestation at which probiotics are discontinued; contraindications to probiotic use; presence of a unit-specific probiotics guideline and parent information leaflets; microbiology department awareness of neonatal probiotic

Confidential: For Review Only

use; and probiotic administration during scenarios when an infant had sepsis, NEC and/or was made nil by mouth. The survey was trialled by the research team which comprised a medical statistician, five neonatal consultants and two senior neonatal trainees and underwent five iterations before the content was finalised. The research team aimed for a minimum 70% response rate to the survey based upon similar targets set for other UK neonatal clinical practice surveys.[12]

The project was introduced at the 2022 Summer meeting of the NeoTRIPS collaborative group. NeoTRIPS is a national, trainee-led, neonatal research network affiliated with the British Association of Perinatal Medicine (BAPM).[13] Regional NeoTRIPS leads for each neonatal ODN were provided with the survey link which was then disseminated to local NeoTRIPS leads for each participating neonatal unit. The survey invitation contained instructions for one survey to be completed per unit by a trainee. Whilst trainee seniority was not captured in the survey, participants were encouraged to discuss responses they were uncertain of (such as whether microbiology were aware) with senior unit clinicians. Survey reminders were sent to the regional NeoTRIPS leads fortnightly during the survey period.

There are 15 neonatal ODNs including 188 units in the UK. Centres caring for newborn infants in the UK are stratified into three levels: level 1 which refers to special care units (SCUs); level 2 or local neonatal units (LNUs); and highly specialised level 3 neonatal intensive care units (NICUs).[14]

Confidential: For Review Only

The survey results were collated in a Microsoft Excel file and data were summarised using descriptive statistics for quantitative responses.

Patient and Public Involvement

In August 2022, focus group sessions with parents, former NICU patients and advocacy groups recruited through the neoWONDER collaborative[15] were held to establish perspectives on the use of routinely collected neonatal data to monitor probiotic efficacy during routine administration. It was explained to participants that the survey report could be instrumental in designing any future studies and the members of this group felt this was important. The focus group was not involved in the design of this survey of clinicians' clinical practice.

Confidential: For Review Only

RESULTS

Survey response rate and probiotic use

Between 13th August 2022 and 31st October 2022, 161/188 (86%) neonatal units completed the survey. There was a NICU response rate of 50/57 (88%), LNU response rate of 72/83 (87%) and SCU response rate of 39/48 (81%). 70/161 (44%) responding units currently use probiotics, constituting 31/50 (62%), 28/72 (39%) and 11/39 (28%) of responding NICUs, LNUs and SCUs, respectively. Figure 1 outlines the response rate and probiotic use by unit designation.

Survey responses for units routinely using probiotics

Table 1 outlines survey responses from the 70 units that confirmed routine probiotic use.

Confidential: For Review Only

Table 1: Survey results for units using probiotics (*n*=70)	
**Survey question**	***n* (%)**
**Probiotic choice**	
Lactobacillus acidophilus NCFM/*Bifidobacterium bifidum* Bb-06/*Bifidobacterium* infantis Bi-26 (Labinic™)	45 (64)
Bifidobacterium infantis Bb-02/*Bifidobacterium lactis* Bb-12/*Streptococcus thermophilus* (ProPrems®)	15 (21)
Bifidobacterium bifidum/Lactobacillus acidophilus (Infloran®)	10 (14)
**Gestation cut-off for probiotic administration**	
≤34 weeks	10 (14)
≤33 weeks	2 (3)
≤32 weeks	57 (81)
≤28 weeks	1 (1)
**When probiotics are started**	
After enteral feeds are commenced	62 (89)
Soon after birth	8 (11)
**Guideline for probiotic administration**	
Yes	64 (91)
No	4 (6)
Unknown	2 (3)
**Microbiology team awareness of neonatal probiotic use**	
Yes	33 (47)
No	2 (3)
Unknown	35 (50)
**Probiotic use during antibiotic administration**	
Continue	55 (79)
Stop	15 (21)
Reduce or modify	0 (0)
**Parent information leaflets**	
Yes	32 (46)
No	30 (43)
Unknown	8 (11)
**Parental refusal of probiotics**	
Yes	5 (7)
No	65 (93)

view Only

<u>Additional responses from units using probiotics</u>

Birthweight:

59/70 (84%) units using probiotics additionally had birthweight as part of their probiotic administration criteria, with 56/59 (95%) starting probiotics in infants ≤1500g; 1/59 (2%) in infants ≤1600g; 1/59 (2%) in infants ≤1800g; and 1/59 (2%) not specifying a birthweight criterion.

When to stop probiotics:

The majority of units [55/70 (79%)] would discontinue probiotics at 34 weeks postmenstrual age, with 1/70 (1%) discontinuing at 32 weeks; 2/70 (3%) at 35 weeks; 2/70 (3%) at 36 weeks; and 2/70 (3%) at term. 3/70 (4%) units would stop probiotics at discharge regardless of postmenstrual age. 5/70 (7%) units would discontinue probiotics at either 34 weeks postmenstrual age or discharge, depending on whether they were commenced due to gestational age or birthweight, respectively.

Contraindications to using probiotics:

31/70 (44%) units listed contraindications to starting probiotics, and where free text comments were provided, these included: intestinal atresia; NEC; sepsis and/or suspected immunocompromise.

Probiotic use during abdominal concerns including necrotising enterocolitis

If enteral feeds are stopped, 59/70 (84%) units would also stop probiotics; 9/70 (13%) would continue and 2/70 (3%) would reduce or modify the dose.

https://mc.manuscriptcentral.com/bmjpo

Confidential: For Review Only

69/70 (99%) units would stop probiotics completely for infants being treated for NEC, and just one unit stated that they would continue. Probiotics would be restarted after an episode of NEC once enteral feeds are reintroduced and/or a set volume of feed is tolerated in 53/69 (77%) units. In 11/69 (16%) units, they would only be restarted based on a consultant and/or surgical team decision. One unit stated they would not restart probiotics after NEC, one would only restart once antibiotics (for NEC) are stopped, and one would only restart once the baby was repatriated to their local hospital from the surgical centre. 2/69 (3%) units were unsure regarding their policy of restarting probiotics after an episode of NEC.

Confidential: For Review Only

Survey responses for units not using probiotics

Table 2 outlines survey responses from the 91 units that do not routinely use probiotics.

Table 2: Survey results for units not using probiotics (*n*=91)

Survey question	*n* (%)
**Intention to introduce routine probiotics within the next 12**	
**months**	24 (26)
Yes	67 (74)
No	
**Parents enquiring regarding probiotic use**	
Yes	18 (20)
No	73 (80)

DISCUSSION

This up-to-date survey with a high response rate represents national practice relating to probiotic use in UK neonatal units. The survey confirms a notable increase in routine probiotic administration, especially in NICUs (now 62%), compared to the last survey in 2018 when this figure was 17%.[8] This may be due to the recent position paper from ESPGHAN recommending specific probiotic strains to reduce rates of NEC (despite low certainty of benefit),[9] which may in turn have increased clinician confidence in using probiotic interventions.

The most frequently used probiotic product in UK neonatal units at the time the survey was conducted was a combination of *Lactobacillus acidophilus*, *Bifidobacterium bifidum* Bb-06 and *Bifidobacterium infantis* Bi-26 (Labinic™). Whilst this probiotic has not been extensively evaluated in large RCTs,[16] it has been reported to show efficacy against NEC, LOS and unadjusted mortality in some studies.[3] Furthermore, it does not meet current ESPGHAN guidelines. The guidelines caution against using some strains of *Lactobacillus acidophilus* due to insufficient safety data relating to the effects of D-lactate production by certain strains of this bacteria.[9]

The second most commonly used combination is *Bifidobacterium infantis*, *Bifidobacterium lactis* Bb-12 and *Streptococcus thermophiles* (ProPrems®). This combination of probiotic bacteria has been evaluated in two RCTs, but neither was powered for NEC as a primary outcome.[1,17] The combination is however endorsed

Confidential: For Review Only

in the ESPGHAN position paper and further RCTs of its efficacy against NEC and mortality in extremely preterm babies are planned (ClinicalTrials.gov: NCT05604846).

Choosing which probiotic to use on a neonatal unit can sometimes be dependent upon availability rather than using products which have robust evidence of benefit. Van den Akker *et al* cautioned against this, stating that *'clinicians may be left using inadequately tested, potentially unsafe and possibly ineffective treatments'*.[7] Furthermore, there is inconsistency in the evidence of benefit in pre- and post-implementation studies using Labinic™ and the combination of *Bifidobacterium bifidum* and *Lactobacillus acidophilus* (Infloran®) in UK neonatal units. Whilst one study reported a significant NEC reduction during epochs after these probiotics were routinely implemented,[3] another more recent study did not show evidence of benefit.[4] Why these differences exist in different geographic locations but within the same country is unclear, but may reflect differences in infants, unit practices or disease categorisation.

Inconsistencies in probiotic efficacy are also recognised across large RCTs. Among the two largest, the ProPrems trial reported a significant reduction in NEC amongst infants randomised to a probiotic combination containing *Bifidobacterium infantis, Streptococcus thermophilus* and *Bifidobacterium lactis*[1]; however, for participants in the PiPS trial, there was no evidence of NEC reduction among infants randomised to *Bifidobacterium breve* BBG-001.[2] Neither trial reported significant reductions in LOS or mortality. Strain selection and probiotic combinations are therefore important and

Confidential: For Review Only

may act differently in different populations. Not all are effective, and none has been shown to definitively reduce all of NEC, LOS and death.

Probiotic safety is frequently highlighted when considering routine probiotic supplementation in preterm infants. Adverse clinical events have mostly taken the form of isolated case reports of bacteraemias and given the large numbers of preterm babies exposed to probiotics in clinical trials, these reports appear infrequently.[18] Probiotic contamination has also been reported and associated with death from fungal contaminants.[19] Using probiotic products that are manufactured to the highest pharmaceutical standard is thus imperative. In this current survey, more than 50% of respondents were unsure if their local microbiology department was aware that probiotics were being used on the neonatal unit. This may represent the responses of more junior members of the team or a failure to educate clinicians about the importance of probiotic implementation surveillance. It also potentially highlights the lack of appreciation/awareness of the importance of involving local laboratory services to ensure there is a system in place to detect adverse events associated with probiotic use. When using live interventions, it is imperative that unit guidelines are designed with input from local microbiology teams so that patient and product safety can be monitored.[20] If they are not, adverse events associated with probiotic use could be under-reported.

Over half of responding units stated they do not currently use probiotics, but we did not specifically ask why this was. Lack of availability of products with clear evidence

https://mc.manuscriptcentral.com/bmjpo

Confidential: For Review Only

of efficacy, conflicting reports from large RCTs, cost, a lack of national consensus guidelines and safety concerns are recognised to contribute to clinician reluctance to implement routine probiotic use. Amongst units not currently using probiotics, 26% (24 units) indicated they intend to start routine probiotic administration within the next year. With the increasing number of units moving towards routine administration, it is important that probiotic efficacy is monitored systematically across the UK's neonatal population. One way this could be achieved is to monitor efficacy using large population databases of routinely-collected electronic patient record data, such as the National Neonatal Research Database (NNRD). To do this, these databases would need to record probiotic use in a manner that allows easy extraction of these data and reliable comparisons of clinical endpoints in babies exposed to probiotics versus those who have not been exposed. During patient and parent focus groups associated with designing this project, participants were unanimously supportive towards maximising the utility of routinely collected data to address some of the uncertainties around probiotic efficacy. Monitoring safety during routine implementation is equally important and utilising systems such as the British Paediatric Surveillance Unit[21] could be one way to capture and report any adverse events associated with probiotic use.

One of the greatest strengths of this study was the high response rate of 86%. This exceeded the target of 70% and demonstrates the willingness of trainees to undertake research. Furthermore, the survey was completed within 12 weeks making results available in a timely and efficient manner. The main limitation of the study was that

Confidential: For Review Only

parts of the survey that allowed free text entries were not always completed (e.g., contraindications to using probiotics).

Confidential: For Review Only

CONCLUSIONS

This survey confirms that an increasing number of UK neonatal units are administering probiotics to preterm babies. As units move towards routine probiotic use, systems to monitor efficacy are needed as some of the probiotics being used have limited evidence of benefit. Evaluation of efficacy could be achieved by interrogating routinely collected national datasets. Robust reporting of any adverse outcomes associated with probiotic use are also needed so that adequate safety monitoring occurs in parallel with routine implementation.

Acknowledgements

We would like to thank all the neonatal units that contributed to this survey.

Contributors

NP, KE, JB, LS, KC, SO, PF and CB were involved in the conception, methodology, writing, reviewing, and editing of the manuscript. NP, KE, and the NeoTRIPS Collaborative Group were involved in data acquisition.

Funding

No formal funding was provided for this study.

Competing Interests

None of the authors has a conflict of interest to declare.

Confidential: For Review Only

Patient consent for publication

Not required.

Ethical approval

Formal ethical approval was not required for this evaluation of service provision. No patient identifiable information was sought or gathered in this survey.

Data availability statement

All data relevant to the study are included in the article or uploaded as supplementary information.

Confidential: For Review Only

REFERENCES

1. Jacobs SE, Tobin JM, Opie GF, et al. Probiotic effects on late-onset sepsis in very preterm infants: a randomized controlled trial. *Pediatrics* 2013;132(6):1055-62. doi: 10.1542/peds.2013-1339 [published Online First: 20131118]
2. Costeloe K, Bowler U, Brocklehurst P, et al. A randomised controlled trial of the probiotic Bifidobacterium breve BBG-001 in preterm babies to prevent sepsis, necrotising enterocolitis and death: the Probiotics in Preterm infantS (PiPS) trial. *Health Technol Assess* 2016;20(66):1-194. doi: 10.3310/hta20660
3. Robertson C, Savva GM, Clapuci R, et al. Incidence of necrotising enterocolitis before and after introducing routine prophylactic Lactobacillus and Bifidobacterium probiotics. *Arch Dis Child Fetal Neonatal Ed* 2020;105(4):380-86. doi: 10.1136/archdischild-2019-317346 [published Online First: 20191030]
4. Granger C, Dermyshi E, Roberts E, et al. Necrotising enterocolitis, late-onset sepsis and mortality after routine probiotic introduction in the UK. *Arch Dis Child Fetal Neonatal Ed* 2022;107(4):352-58. doi: 10.1136/archdischild-2021-322252 [published Online First: 20210825]
5. Sharif S, Meader N, Oddie SJ, et al. Probiotics to prevent necrotising enterocolitis in very preterm or very low birth weight infants. *Cochrane Database Syst Rev* 2020;10(10):CD005496. doi: 10.1002/14651858.CD005496.pub5 [published Online First: 20201015]
6. Jarrett P, Meczner A, Costeloe K, et al. Historical aspects of probiotic use to prevent necrotising enterocolitis in preterm babies. *Early Hum Dev* 2019;135:51-57. doi: 10.1016/j.earlhumdev.2019.05.015 [published Online First: 20190529]
7. van den Akker CHP, van Goudoever JB, Szajewska H, et al. Probiotics for Preterm Infants: A Strain-Specific Systematic Review and Network Meta-analysis. *J Pediatr Gastroenterol Nutr* 2018;67(1):103-22. doi: 10.1097/MPG.0000000000001897
8. Duffield SD, Clarke P. Current use of probiotics to prevent necrotising enterocolitis. *Arch Dis Child Fetal Neonatal Ed* 2019;104(2):F228. doi: 10.1136/archdischild-2018-316199 [published Online First: 20181121]
9. van den Akker CHP, van Goudoever JB, Shamir R, et al. Probiotics and Preterm Infants: A Position Paper by the European Society for Paediatric Gastroenterology Hepatology and Nutrition Committee on Nutrition and the European Society for Paediatric Gastroenterology Hepatology and Nutrition Working Group for Probiotics and Prebiotics. *J Pediatr Gastroenterol Nutr* 2020;70(5):664-80. doi: 10.1097/MPG.0000000000002655
10. NECUK. www.necuk.org.uk: NEC UK; 2018 [accessed April 4th 2023.
11. Google. https://docs.google.com/forms: Google Forms; [accessed April 4th 2023.
12. Dorling J, Tume L, Arch B, et al. Gastric residual volume measurement in British neonatal intensive care units: a survey of practice. *BMJ Paediatr Open* 2020;4(1):e000601. doi: 10.1136/bmjpo-2019-000601 [published Online First: 20200807]
13. NeoTRIPS. https://neotrips.org: NeoTRIPS; 2022 [accessed April 4th 2023.
14. NHS. Toolkit for high quality neonatal services. London, UK. 2009
15. neoWONDER. https://www.neowonder.org.uk/: neoWONDER; 2022 [accessed April 4th 2023.
16. Sowden M, van Niekerk E, Bulabula ANH, et al. Effect of a Multi-Strain Probiotic on Growth and Time to Reach Full Feeds in Preterm Neonates. *Nutrients* 2022;14(21) doi: 10.3390/nu14214658 [published Online First: 20221103]

Confidential: For Review Only

17. Bin-Nun A, Bromiker R, Wilschanski M, et al. Oral probiotics prevent necrotizing enterocolitis in very low birth weight neonates. *J Pediatr* 2005;147(2):192-6. doi: 10.1016/j.jpeds.2005.03.054
18. Fleming PF, Berrington JE, Jacobs SE. Addressing safety concerns of probiotic use in preterm babies. *Early Hum Dev* 2019;135:72-74. doi: 10.1016/j.earlhumdev.2019.05.016 [published Online First: 20190530]
19. Vallabhaneni S, Walker TA, Lockhart SR, et al. Notes from the field: Fatal gastrointestinal mucormycosis in a premature infant associated with a contaminated dietary supplement--Connecticut, 2014. *MMWR Morb Mortal Wkly Rep* 2015;64(6):155-6.
20. Barbian ME, Buckle R, Denning PW, et al. To start or not: Factors to consider when implementing routine probiotic use in the NICU. *Early Hum Dev* 2019;135:66-71. doi: 10.1016/j.earlhumdev.2019.05.009 [published Online First: 20190610]
21. BPSU. https://bpsu.org.uk: Surveillance of Rare Childhood Disease 2019 [accessed April 4th 2023.

Confidential: For Review Only

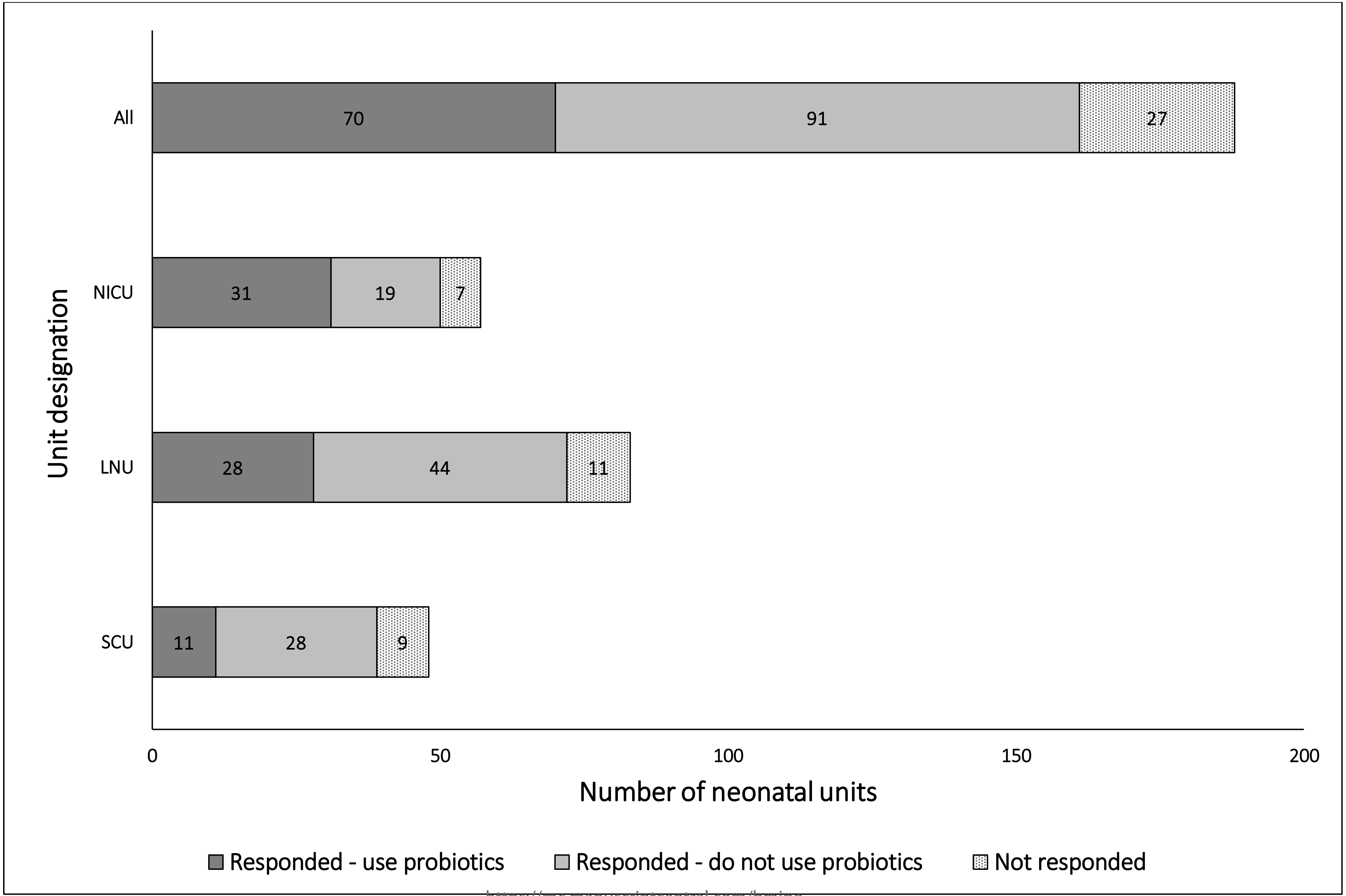
Unit designation
All
NICU
LNU
SCU
70
91
27
31
19
7
28
44
11
11
28
9
0
50
100
150
200
Number of neonatal units
Responded - use probiotics
Responded - do not use probiotics
Not responded

Evaluating the use of probiotics in UK neonatal units: a national, trainee-led survey

This is a national, trainee-led survey evaluating the use of probiotics in all UK neonatal units, conducted as part of NeoTRIPS.

NeoTRIPS is a national neonatal study group, using the power of trainees to improve neonatal care through large scale quality improvement and research projects.

Please ensure that only 1 trainee responds per neonatal unit.

* Required

1. What is the name of your neonatal operational delivery network (ODN)? *

   Mark only one oval.

   - East Midlands Neonatal ODN
   - East of England Neonatal ODN
   - Neonatal Network Northern Ireland
   - North Central and East London Neonatal Network
   - North West London Neonatal ODN
   - North West Neonatal ODN
   - Northern Neonatal Network
   - Scottish Perinatal Network
   - South East Coast Neonatal Network
   - South London Network
   - South West Neonatal Network
   - Thames Valley & Wessex Neonatal Network
   - Wales Maternity and Neonatal Network
   - West Midlands Neonatal ODN
   - Yorkshire & Humber Neonatal ODN

2. What is the name of your neonatal unit/local hospital? *

3. What is the level of your neonatal unit? *

   Mark only one oval.

   - Level 1
   - Level 2
   - Level 3

4. Does your neonatal unit currently use probiotics? *

Mark only one oval.

- Yes *Skip to question 5*
- No *Skip to question 20*
- Don't know

Neonatal units utilising probiotics

5. What probiotic do you currently use? *

Mark only one oval.

- ProPrems
- Infloran
- Labinic
- Other: ____________________

6. What gestational age group receive probiotics? *

____

7. Does birthweight influence probiotic use? *

Mark only one oval.

- Yes
- No

8. If birthweight does influence probiotic use, please explain:

____
____
____
____
____

9. Does your unit have any circumstances in which probiotic administration is contraindicated e.g. congenital bowel atresia? *

Mark only one oval.

- Yes
- No
- Other: ____________

10. When does your unit start probiotics? *

Mark only one oval.

- At birth
- After commencing enteral feeds
- Once the baby reaches full enteral feeds
- Other: ____________

11. At what point would your unit discontinue probiotic supplementation e.g. at how many weeks postmenstrual age (PMA)? *

____

12. Does your unit have a probiotics guideline? *

Mark only one oval.

- Yes
- No
- Don't know

13. Is your Microbiology lab aware of your unit using probiotics? *

Mark only one oval.

- Yes
- No
- Don't know

14. If a baby is on IV antibiotics for suspected sepsis, is your unit policy to: *

Mark only one oval.

- ( ) Stop probiotics completely
- ( ) Reduce/modify the dosage
- ( ) Continue with probiotics

15. If a baby's enteral feeds are withheld, does your unit: *

Mark only one oval.

- ( ) Stop probiotic supplementation whilst feeds are withheld
- ( ) Reduce/modify the dosage of the probiotics whilst feeds are withheld
- ( ) Continue probiotics whilst feeds are withheld

16. If you are treating a baby for necrotising enterocolitis, does your unit: *

Mark only one oval.

- ( ) Stop probiotics supplementation whilst feeds are withheld
- ( ) Reduce/modify the dosage of the probiotics whilst feeds are withheld
- ( ) Continue probiotics whilst feeds are withheld

17. Following treatment of necrotising enterocolitis, when are probiotics restarted? *

Mark only one oval.

- ( ) When antibiotics are completed
- ( ) When enteral feeds are restarted
- ( ) When the baby reaches full enteral feeds
- ( ) Never
- ( ) Other: ______________________

18. Does your unit provide a probiotic parent information leaflet? *

Mark only one oval.

- Yes
- No
- Don't know

19. Has your unit experienced any parental refusal for probiotics? *

Mark only one oval.

- Yes
- No

Neonatal units not utilising probiotics

20. Have you encountered parents enquiring about probiotics? *

Mark only one oval.

- Yes
- No

21. Is your unit planning to use probiotics within the next 12 months? *

Mark only one oval.

- Yes
- No

This content is neither created nor endorsed by Google.

Google Forms
